# How People React to the Termination of an Intimate Relationship: An Exploratory Mixed-Methods Study

**DOI:** 10.1177/14747049241312231

**Published:** 2025-01-12

**Authors:** Menelaos Apostolou, Isaias Taliadoros, Timo Juhani Lajunen

**Affiliations:** 1 Department of Social Sciences, 121343 University of Nicosia, Nicosia, Cyprus; 2 Department of Psychology, 8018 Norwegean University of Science and Technology, Trondheim, Norway

**Keywords:** termination of a relationship, intimate relationships, negative emotions

## Abstract

Intimate relationships frequently come to an end, and in the current research, we have endeavored to examine how individuals would potentially react in the scenario where their intimate partner decides to terminate a relationship they wish to continue. More specifically, employing open-ended questionnaires on a sample of 219 Greek-speaking participants, we identified 79 possible reactions. Subsequently, using close-ended questionnaires on a sample of 442 Greek-speaking participants, we categorized these reactions into 13 broad factors. Participants indicated that they were more likely to feel sadness, inquire of their departing partners why they wish to end the relationship, and attempt to divert their thoughts elsewhere to avoid dwelling on the end of the relationship. Men indicated a higher likelihood than women to seek revenge sex, although significant sex differences were not observed in other reactions. Furthermore, we classified these 13 factors into three broader domains. The highest-rated domain was “Accept and forget,” followed by “Sadness and depression,” and “Physical and psychological aggression.” These findings could enable us to gain a better understanding of the process of relationship dissolution, and could potentially be employed to identify and prevent reactions that may have harmful repercussions for the individuals involved in the relationship.

## Introduction

Forming lasting intimate relationships constitutes a human universal (Buss, 2016; Fisher, 2017). However, intimate relationships do not always endure a lifetime and often end as the relatively high rates of divorce testify (Raley & Sweeney, 2020). The current research aims to examine how people react to the termination of a romantic relationship that they had hoped would last. These reactions can be best understood within an evolutionary theoretical framework, which we will discuss next.

### Reactions to the Termination of an Intimate Relationship

Forming intimate relationships is of outmost evolutionary importance for several reasons. To begin with, humans are a sexually reproducing species, which means that individuals require access to the reproductive capacity of the opposite sex to procreate (Muehlenbein, 2015). Moreover, children necessitate substantial parental investment to reach sexual maturity—a burden that would be challenging for a single parent to bear (Hrdy, 2011). Additionally, an intimate partner serves as a source of material and emotional support, particularly during times of need (Apostolou, 2022).

When an intimate relationship ends, we can reasonably assume that the party leaving it does so in order to increase its survival and reproductive success, or fitness—one possible reason being that it has found a higher mate value mate. On the other hand, the party who wants to stay in the relationship does so, because it considers it fitness-increasing. Thus, the termination of an intimate relationship by one partner would be associated with a decrease in the fitness of the other partner. Furthermore, forming and maintaining an intimate relationship require substantial investment, such as dedicating time and resources to enhance one's appeal as a mate, providing resources to support and satisfy the partner, and allocating resources to protect the partner (Apostolou et al., 2023; Buss, 2002). When a relationship ends, this investment is forfeited, further reducing the fitness of the party not intending to terminate the relationship.

Overall, the scenario in which individuals find themselves in a relationship that their partners end potentially involves a substantial fitness cost. This cost would result in strong selection pressures shaping mechanisms or adaptations to prevent its recurrence. Such mechanisms are emotions that we will discuss next.

### Emotions and the Termination of a Relationship

Emotions constitute adaptations that regulate human behavior by motivating fitness-increasing actions (Tooby & Cosmides, 2008). For example, when individuals are injured, they experience pain, which motivates them to take corrective actions, such as visiting a doctor, to alleviate it. To use another example, social exclusion can lead to sadness and anger that in turn, could lead to attempts to fortify relational needs (Williams, 2007). We argue that selection forces have shaped emotional mechanisms to generate responses that reduce the likelihood of individuals finding themselves in situations where their intimate partners leave them (see also Leary, 2015).

To begin with, we argue that a scenario in which one partner desires to end the relationship while the other wishes to continue it, would elicit strong negative emotions in the latter, such as sadness and despair (see also Boelen & Reijntjes, 2009). These emotional responses would potentially increase fitness through different avenues. In particular, they would motivate people to make efforts to salvage the relationship, for instance, by seeking another chance after making necessary changes. More importantly, the emotional pain would prompt individuals to reflect on the reasons for the relationship's failure and take action to avoid similar situations in the future. The emotional distress would also serve as a deterrent, motivating individuals to make more judicious mate choices in the future to prevent similar outcomes: When engaging in mate-seeking, they would remember the negative emotions caused by the termination of previous relationships, leading them to direct their mating efforts toward partners less likely to leave them.

Furthermore, we hypothesize that another emotional response would be anger, which would potentially lead to psychological and physical aggression (see also Buss, 2021; Buss & Duntley, 2006). This response might increase fitness by deterring an intimate partner from ending the relationship: Individuals may fear potential harm from their partners, which would discourage them from terminating the relationship. This outcome is more likely in the pre-industrial context, and thus, throughout most of human evolution, where individual rights are not as well protected, and violence between intimate partners is more tolerated or even expected (Campbell, 1973). Additionally, anger constitutes a negative emotion; thus, experiencing it is unpleasant, encouraging individuals to reflect on what went wrong and make better choices in the future to avoid such experience.

The emotional responses described above are relevant to situations where one partner perceives the relationship as fitness-increasing while the other does not, leading to the dissolution of the relationship. However, this is not the only pathway to relationship termination. In some cases, the relationship may be fitness-decreasing for both partners who may mutually agree to end it. Additionally, there may be instances where one partner recognizes the negative nature of the relationship but hesitates to end it due to concerns about the other partner's reactions. In these scenarios, the eventual termination of the relationship can lead to positive emotions such as happiness or relief for both parties. We will now proceed to discuss the impact of the termination of an intimate relationship on self-esteem.

### Self-esteem and the End of an Intimate Relationship

Self-esteem, defined as one's perceived worthiness as a person (Baumeister, 1993), is another mechanism likely to play a role in the termination of a desirable relationship. In particular, it has been argued that it is an evolved mechanism providing individuals with adaptively relevant feedback about their position in the social world (Barkow, 1980; Kenrick et al., 2010; Leary, 1999). In particular, the so called sociometer theory of self-esteem argues that it constitutes a psychological gauge of the degree to which people perceive that they are relationally valued and socially accepted by other people (Leary, 2012). In the domain of mating, people seek partners who are similar to them in several dimensions, such as looks, social status, and financial capacity (Luo, 2017). Self-esteem informs individuals about their worthiness as potential mates, subsequently guiding their mating efforts toward attracting partners with similar traits.

Yet, one's worthiness as a mate can change with circumstances and age. For instance, securing a high-status job can increase an individual's worthiness, while job loss can decrease it. The termination of an intimate relationship can potentially provide information about the staying partner's worthiness as a mate. For example, it is possible that the staying partner's worthiness has decreased, motivating the departing partner to seek another mate. In such cases, the termination of the relationship can negatively affect the self-esteem of the staying partner. Lower self-esteem signals to the latter that they possess a mate worthiness lower than they previously believed, prompting them to seek future partners closer to their current worthiness. In total, we hypothesize that in scenarios where one party terminates the relationship while the other desires to maintain it, negative emotions such as sadness, depressive mood, loneliness, and despair, along with anger and reduced self-esteem, could be triggered in the staying partner. Existing literature provides some support for these predictions.

### Current Literature

An intimate partner ending the relationship may be interpreted as rejecting the other partner, and there is a considerable body of literature on how people respond to rejection. To begin with, studies have consistently found that being rejected by a stranger is associated with negative emotions and a reduction in self-esteem (for comprehensive reviews, see Abrams et al., 2005; Hartgerink et al., 2015; Smart Richman & Leary, 2009; Williams, 2007; Williams et al., 2005). While most research indicates that negative reactions from others often evoke emotions such as hurt and sadness (Vangelisti et al., 2005), some studies have reported emotional numbing as an alternative response (Twenge et al., 2003). Additionally, individuals who experience criticism or ostracism often display anger and aggressive behaviors (Leary et al., 2006; Warburton et al., 2006 see also Williams, 2007). In one study, Kross et al. (2011) investigated the neural mechanisms underlying social rejection and discovered that it activates the brain's pain matrix—the same areas of the brain activated by physical pain. Crocker and Park (2004) investigated the effects of rejection and found that it can lead to a decrease in self-esteem, particularly among individuals who are already grappling with low self-esteem.

In the context of intimate relationships, Baumeister et al. (1993) examined the impact of unrequited love. More specifically, they collected stories on unrequited love from samples of students in the United States and compared the accounts of rejectors and would-be lovers. They found that would-be lovers’ accounts typically involved negative emotions and damage to self-esteem. Davis et al. (2003) attempted to examine whether attachment styles predicted responses to break up, and for this purpose, guided by their theoretical perspective, they constructed an instrument to measure these responses. The authors conceptually divided the instrument into 10 reactions, namely “wanting/trying to get the person back,” “sexual arousal/desire,” “anger/hostility/revenge,” “physically hurting partner,” “preoccupation,” “interference with exploration,” “physical and emotional distress,” “lost interest in sex,” “self-blame,” and “partner blame.” In a different study Freedman et al. (2022) interviewed 80 participants who had been ghosted (i.e., their partners unilaterally ended the relationship by ceasing communication) in previous romantic relationships. Their responses indicated sadness and hurt feelings as well as a negative effect to their self-esteem. Several studies have also reported a decline in mental health following the termination of an intimate relationship (for a comprehensive review, see Mirsu-Paun & Oliver, 2017).

### The Current Study

Existing literature provides substantial evidence that rejection is associated with negative emotions, anger, and diminished self-esteem. Nevertheless, a study is needed that would attempt to identify an inclusive list of emotional and behavior reactions to the termination of a relationship people wish to continue. Such a study is necessary because reactions in the domain of mating may differ from those in other domains, such as friendship. To the best of our knowledge, there was only one such attempt by Davis et al. (2003) who nominated 10 possible reactions. However, these reactions were identified conceptually and not empirically. This task requires identifying an inclusive list of reactions empirically, using qualitative research methods, and using quantitative research methods and appropriate date reduction techniques such as factor analysis, classifying these reactions into broader categories. Consequently, the current study was designed to address this gap in the literature by employing a mixed-methods approach. Specifically, Study 1 utilized qualitative research methods to compile a comprehensive list of reactions to the termination of an intimate relationship, while Study 2 employed quantitative research methods to categorize these reactions into broader factors and domains.

Based on our theoretical framework and the existing literature on rejection, we hypothesize that reactions to the termination of an intimate relationship will cluster into factors and domains reflecting negative emotions, anger, and a negative impact on self-esteem. Nevertheless, given the complexity of the phenomenon and the limited research in this area, we acknowledge that we cannot predict all possible reactions to the termination of a relationship, and thus, we consider our study exploratory. Additionally, considering different evolutionary trajectories, it is well-documented that, on average, men possess greater physical strength and tend to exhibit more aggression than women (Buss, 2021). Consequently, we predict that men would be more inclined than women to engage in aggressive behavior in response to the termination of an intimate relationship. Furthermore, apart from sex, in the current study, we will examine age and relationship status effects without making specific directional hypotheses.

## Study 1

The purpose of the current study is to identify the different reactions to the termination of a desirable relationship using qualitative research methods.

## Method

### Participants

The research project was devised and executed at a private university situated in the Republic of Cyprus, and it secured ethical approval from the institution's ethics review board. In terms of participant recruitment, we disseminated the study's link across various social media platforms (namely Facebook, Instagram, and X) and distributed it to students, urging them to share it within their circles of friends and family. The sole criterion for participation was attaining a minimum age of 18 years. To bolster the study's validity, we excluded responses from eight participants who disclosed that they were presently single and lacked prior experience in intimate relationships. Our dataset, therefore, comprises data from a cohort of 219 Greek-speaking individuals (111 women, 108 men). Women had an average age of 31.2 years (*SD *= 9.2), while men had an average age of 32.2 years (*SD *= 10.4). Additionally, 52.3% of the participants reported being in an intimate relationship, 38.7% were not currently in an intimate relationship, and 9% designated their relationship status as “other.”

### Materials

We employed an act nomination approach used in previous research (e.g., Apostolou, 2013; Buss & Craik, 1980). The study was conducted online in Greek and was designed using Google Forms. The questionnaire was divided into two parts. In the first part, participants were presented with a scenario in which they were in an intimate relationship that they wished to continue, but their partners expressed a desire to end the relationship. Subsequently, they were asked to indicate their potential reactions to this situation. In the second part, demographic information was collected, including gender, age, and relationship status (currently not in an intimate relationship, currently in an intimate relationship, or “other”). Additionally, participants were asked to indicate whether they had previously been in an intimate relationship (Yes/No).

## Analysis and Results

The open-ended questionnaire was designed to enable participants to provide a list of different possible reactions to the termination of an intimate relationship. Our aim was to refine these answers and create an inclusive list of these reactions. For this purpose, the following methodology was employed: Two postgraduate students, one male and one female, were enlisted to independently process the dataset. The research assistants were informed that the purpose of the study was to identify the different reactions to the termination of an intimate relationship, but they were blind to the specific hypotheses. Each research assistant was assigned the responsibility of reviewing respondents’ answers and assembling a list of reactions associated with the termination of the relationship. Whenever a unique reaction was encountered, it was incorporated into the compilation. Responses containing unclear or convoluted language were excluded, and in cases where multiple reactions closely resembled each other, only one was retained. Upon processing approximately 30% of the responses, the assistants engaged in a discussion regarding their findings, involving one of the authors. Subsequently, they continued processing the remaining responses. Following the completion of the analysis, the assistants cross-referenced their respective lists and, with input from one of the authors, reached a consensus on the final list of reaction. In total, 79 reactions were identified. Not all the 79 items were retained in the analysis performed in Study 2; the ones retained are presented in Table 1 and the ones removed are presented in Appendix.

**Table 1.** The reactions identified in Study 1 and results of exploratory factor analysis in Study 2.

Factors Disadvantages	Factor loadings	Cronbach's α
Change his/her mind		.94
I would tell him/her not to make rash decisions and to think again	.810	
I would tell him/her to take some time to think it over	.761	
I would tell him/her that it is a shame to destroy everything we have built	.761	
I would ask him/her if there is room to change his mind	.721	
I would ask him/her if there is anything we can do to avoid getting there	.683	
I would tell him/her that I love him/her and I don't want to break up	.628	
I would ask him/her to give me a second chance	.625	
I would try to change his/her mind	.623	
I would ask him/her to discuss it	.496	
I would tell him/her that I am willing to make changes to make our relationship work	.495	
I would ask him/her if it is because of some problem that could be solved	.457	
I would ask him/her to consider the consequences of our separation for other people (family, children, friends)	.449	
I would show him/her how saddened I am by his/her decision	.443	
If we had, I would ask him to think about our children	.416	
Cut all contact		.81
I would cut off all contact with him/her	.741	
I would never speak to him/her again	.724	
I would block him/her on social media	.620	
I would disappear from his/her life	.615	
I would throw away/destroy anything that reminded me of him/her	.380	
I would try to get him/her completely out of my mind	.328	
Acceptance		.80
I would respect his/her decision	.737	
I would accept his/her decision	.716	
I would accept it with dignity	.698	
I would wish him/her the best and move on	.613	
I would try to keep calm	.533	
I would accept his/her decision without doing anything	.484	
I would try to convince myself that it is better this way	.331	
I would self-criticize why we reached this result	.321	
Feel depressed		.91
I would get depressed	−.761	
I would shut myself up	−.680	
I wouldn't be in the mood for anything	−.606	
I would be psychologically devastated	−.555	
I would feel insecure	−.486	
I would go crazy	−.429	
I would have a hard time accepting that	−.429	
I would blame myself	−.411	
I would be disappointed	−.375	
I would be shocked	−.346	
I would be upset	−.327	
Become aggressive		.72
I would slap him/her	.720	
I would become violent toward him/her	.672	
I would swear at him/het	.548	
I would throw his/her things on the street	.397	
I would spit on him/her	.389	
Occupy my mind with something else		.67
I would go out often to occupy my mind with something else	−.753	
I would do activities to occupy my mind with something else	−.658	
I would through in my work so I wouldn’t have to think about it	−.481	
I would turn to my friends for support	−.407	
Revenge sex		.70
I would have sex with others and make sure he/she found out	.703	
I would seek to have sex/relationship with someone he/she knows	.703	
I would seek to get into another relationship quickly	.414	
I would look for ways to get revenge on him/her	.371	
Ask why		.79
I would ask him/her to explain why	.679	
I would demand to know the reason	.628	
I would ask him/her if there is another person	.563	
I would try to understand the reasons why this happened	.482	
		.71
Seek the assistance of a psychologist		
I would seek the help of a psychologist to overcome it	−.823	
I would suggest to him/her that we see a psychologist	−.646	
Become angry		.85
I would be angry	.860	
I would get irritated	.824	
I would feel rage	.735	
I would feel betrayed	.336	
Feel sad		.84
I would be sad	−.533	
I would feel down	−.444	
I would be hurt	−.418	
Threaten suicide		.56
I would threaten him/her to kill myself	.669	
I would hurt myself	.506	
Spy on him/her		.65
I would ask our mutual friends about his/her movements	.424	
I would monitor his/her social media movements	.400	

## Study 2

The purpose of the current study was to classify the 79 reactions identified in Study 2 into broad categories and broader domains using quantitative research methods.

## Method

### Participants

Participants were recruited using the same process as in Study 1. However, the study was forwarded to different students than those in Study 1. The sole prerequisite being a minimum age of 18 years. To enhance the validity of our study, we excluded the responses of 13 participants who indicated that they were presently single and had no prior experience in intimate relationships. Thus, our final sample for analysis encompassed 442 Greek-speaking participants (258 women, 182 men, and two participants who did not specify their sex). The average age of women was 31.4 years (*SD *= 10.7), while men had an average age of 35.1 years (*SD *= 12.2). Moreover, 47.3% of the participants indicated they were currently in an intimate relationship, 46.6% reported not being in an intimate relationship, and 6.1% specified their relationship status as “other.” All data are available here: https://osf.io/rx34t/?view_only=87829e8346524388a74802922730ca8b

### Materials

The study was conducted online in Greek, designed using Google Forms, and comprised two parts. In the first part, participants were presented with the following scenario: “You are in a romantic relationship that you would like to continue, and your partner tells you that he/she wants to break up. Please indicate how likely you are to react in the following ways using this scale: 1 = *not at all likely to react this way*, 5 = *very likely to react this way*.” Subsequently, participants were provided with the 79 reactions identified in Study 1. The order of presentation of each reaction was randomized across participants. In the second part, demographic information was collected, including sex, age, and relationship status (currently not in an intimate relationship, currently in an intimate relationship, “other”). Moreover, participants were asked to indicate if they had been in an intimate relationship before (Yes/No).

### Data Analysis

Most likely, the 79 reactions identified in Study 1, reflect different expressions of more fundamental reactions to the termination of an intimate relationship, and factor analysis is required to classify them into broader groupings. Accordingly, we employed exploratory factor analysis, utilizing the principal axis factoring method for factor extraction and direct oblimin rotation. We employed the Kaiser criterion to determine the number of factors to retain, opting to keep all factors with eigenvalues exceeding one. Additionally, we employed a cutoff point of 0.30 to determine the number of items to retain in each factor (see Field, 2017). This procedure was repeated in order to classify the identified factors into broader domains. Factor analysis classified reactions into broader factors, which means that the different reactions should not be treated independently but as part of a group (see also supplemental material for a summary of the instrument constructed). Thus, in order to identify significant effects of variables such as sex, we need to employ multivariate tests that allow more than one dependent variable.

Accordingly, we conducted a series of MANCOVA tests, using each identified factor as the dependent variable. In particular, the acts composing each factor entered as the dependent variables, participants’ sex and relationship status were incorporated as categorical independent variables, and age was incorporated as a continuous independent variable. Thus, in the next section, we will present first the results of factor analysis, and subsequently we will present the results of MANCOVA analysis on the effects of our choice of independent factor on the factors extracted. Finally, we will present the results of the second order factor analysis, where we have conducted further factor analysis to find out whether the original factors can be classified into broader domains.

## Results

### Factor Structure

Initial factor analysis indicated that some items had factor loadings below the 0.30 cutoff point. To address this issue, we conducted the analysis by excluding the item with the lowest factor loading and repeated the procedure until a factor solution emerged in which all items had loadings of at least 0.30. In total, 10 items were removed, and they are listed in the Appendix. The remaining 69 items were subsequently classified into 13 factors (Table 1). The KMO statistic was .93, indicating that our sample size was highly suitable for factor analysis. The internal consistency, measured by Cronbach's alpha, ranged from .56 to .94.

The first reaction to emerge was “Change his/her mind,” in which participants would attempt to persuade their partners to reconsider by encouraging them to think it over, avoid hasty decisions, express regret at the prospect of losing what they had built together, and express their love while requesting a second chance. The second reaction to emerge was “Cut all contact,” in which participants would sever all communication with their departing partners by not talking to or meeting them, and by blocking them on social media. In the “Accept” reaction, participants would try to accept their partners’ decision, maintain composure, show dignity, and wish them the best. In the “Feel depressed” reaction, participants indicated they would experience depression, emotional shutdown, psychological devastation, insecurity, and difficulty accepting the end of the relationship. Furthermore, they would “Become aggressive,” engaging in actions such as slapping their departing partners, using offensive language, spitting, and discarding their belongings on the street.

Moreover, in the “Occupy my mind with something else” reaction, participants would attempt to divert their thoughts from the breakup by going out more often, engaging in activities, and focusing on their work. Another reaction would be “Revenge sex,” where participants would seek revenge on their departing partners by engaging in sexual activities with others, including individuals known to their partners. In the “Ask why” reaction, participants would try to understand why the relationship failed by asking or demanding reasons from their departing partners, including whether there was involvement with another individual. Participants also reported “Seeking the assistance of a psychologist” to help them cope with the breakup or attending therapy sessions with their partners, presumably in an effort to save the relationship.

Additionally, participants reported that they would “Become angry,” feel irritated, and experience a sense of betrayal. They indicated further that the termination of the relationship would make them “Feel sad” and hurt. Some even mentioned they would “Threaten suicide” or self-harm. Finally, in the “Spy on him/her” reaction, participants would inquire with mutual friends and utilize social media to gather information about the whereabouts of their departing partners.

To examine which reactions participants considered more likely and less likely, we calculated the means and standard deviations for each reaction and arranged them hierarchically in Table 2. At the top of the hierarchy was “Feel sad,” followed by “Ask why” and “Occupy my mind with something else” reactions. At the bottom of the hierarchy were “Revenge sex,” “Become aggressive,” and “Threaten suicide” reactions. Additionally, we calculated the number of participants whose mean scores were above “3” for each factor. Given our scale, the percentage indicates how many participants were likely to react in a specific way. This analysis is important because the means can be misleading in terms of the frequency of specific reactions. For example, consider an extreme case where half of the participants do not exhibit a particular reaction at all, while the other half exhibits it very frequently. In this scenario, the mean would be around three, obscuring the fact that half of the participants are highly likely to exhibit that reaction.

**Table 2.** Mean scores, sex, age, and relationship status effects in Study 2.

	Overall		Women	Men	Sex		Age		*Relationship status*	
Factors	%	Mean (SD)	Mean (SD)	Mean (SD)	*p-*value	η_p_*^2^*	*p-*value	η_p_*^2^*	*p-*value	η_p_*^2^*
Feel sad	92.3	4.49 (0.74)	4.53 (0.75)	4.44 (0.71)	.695	.004	.810	.002	.735	.004
Ask why	84.8	4.12 (0.94)	4.09 (0.94)	4.16 (0.95)	.136	.017	(−) .003	.038	.202	.013
Occupy my mind with something else	81.1	3.88 (0.86)	3.94 (0.83)	3.79 (0.89)	.198	.015	(−) < .001	.055	.200	.013
Acceptance	75.3	3.60 (0.80)	3.59 (0.77)	3.61 (0.83)	.077	.055	(+) .002	.057	.190	.025
Become angry	67.1	3.59 (1.10)	3.72 (1.06)	3.42 (1.13)	.473	.009	(+) .001	.047	.448	.010
Feel depressed	67.4	3.46 (0.93)	3.49 (0.94)	3.43 (0.93)	.044	.049	(−) .016	.057	.602	.024
Change his/her mind	60.9	3.29 (1.06)	3.17 (1.05)	3.46 (1.06)	.014	.068	(−) < .001	.106	.497	.034
Cut all contact	46.7	3.04 (0.99)	3.04 (0.96)	3.05 (1.04)	.058	.030	(+) .007	.043	.433	.015
Spy on him/her	39.5	2.90 (1.22)	2.91 (1.18)	2.90 (1.28)	.142	.010	(−) .034	.016	.069	.011
Seek the assistance of a psychologist	28.6	2.48 (1.30)	2.65 (1.28)	2.25 (1.30)	.135	.010	.211	.008	.384	.005
Revenge sex	5.7	1.63 (0.77)	1.48 (0.67)	1.83 (0.86)	<.001	.051	(−) .016	.030	.090	.017
Become aggressive	4.1	1.54 (0.70)	1.62 (0.73)	1.45 (0.63)	.041	.028	.222	.017	.975	.004
Threaten suicide	1.8	1.24 (0.57)	1.22 (0.52)	1.27 (0.63)	.142	.010	(−) .034	.016	.069	.011

*Note.* The signs in parenthesis indicate the direction of the relationship.

The “%” column reports the percentage of participants who had a mean score above “3” in each factor.

From Table 2, we can observe that approximately 92% of the participants indicated that they were likely to feel sad, while only 1.8% of the participants indicated that they were likely to threaten suicide. Furthermore, we calculated how many participants indicated mean scores above “3” for zero, one, two, up to 13 reactions. Note that the maximum was 13 because we had 13 overarching reactions. The results are presented in Graph 1, where it can be seen that most participants indicated that they would be more likely to experience between five and nine reactions, with “7” and “8” being the most common reaction numbers.

### Effects of Sex, Age, Relationship's Status

The analysis encompassed 13 different MANCOVA tests. Consequently, a Bonferroni correction was applied to mitigate alpha inflation, setting the alpha level at .004 (.05/13). Accordingly, *p*-values exceeding this threshold should not be considered statistically significant. As shown in Table 2, a significant main effect of sex was observed only in the “Revenge sex” reaction, where men reported higher scores than women, with the effect size being moderate. Additionally, there was a significant main effect of age on several reactions. As indicated by the effect size, the largest effect was observed for the “Change his/her mind” reaction, followed by the “Feel depressed” and “Acceptance” reactions. For the former two, the regression coefficient was negative, suggesting that older participants tended to provide lower scores. Conversely, for the “Acceptance” reaction, the regression coefficient was positive, indicating that older participants tended to give higher scores. Finally, there was no significant main effect of relationship status on any of the identified reactions.

### Second-Order Factor Structure

Second-order exploratory factor analysis was conducted on the mean scores of the 13 identified factors using the full sample, resulting in a three-domain solution, as presented in Table 3. Within the “Sadness and depression” domain, the highest loading was observed for feelings of sadness and depression, followed by attempts to change the departing partner's mind and inquiries into the reasons behind their desire to end the relationship. In the “Physical and psychological aggression” domain, factors such as becoming aggressive, feeling anger, threatening suicide, and seeking revenge sex exhibited significant loadings. Within the “Accept and forget” domain, factors like cutting all contact, acceptance, and diverting one's attention from the breakup were prominent. Furthermore, we calculated the means and standard deviations for each domain. The highest mean score was found for the “Accept and forget” domain (*M *= 3.51, *SD *= 0.63), followed by the “Negative emotions” domain (*M *= 3.48, *SD *= 0.33), and the “Physical and psychological aggression” domain (*M *= 3.36, *SD *= 0.94).

**Table 3.** The domain structure in Study 2.

Factors Disadvantages	Factor loadings
Sadness and depression	
Feel sad	.796
Feel depressed	.794
Change his/her mind	.727
Ask why	.680
Spy on him/her	.462
Seek the assistance of a psychologist	.418
Physical and psychological aggression	
Become aggressive	.728
Revenge sex	.611
Become angry	.450
Threaten suicide	.436
Accept and forget	
Cut all contact	.638
Acceptance	.520
Occupy my mind with something else	.435

## Discussion

Using qualitative research methods, we identified 79 possible reactions to the termination of a desirable relationship. Employing quantitative research methods, we categorized these reactions into 13 broad factors. Participants indicated that they were more likely to feel sad, inquire why their partners wanted to end the relationship, and attempt to divert their attention from the breakup. Men indicated a greater likelihood than women to seek revenge sex, but no significant sex differences were found in other reactions. Additionally, we grouped the 13 factors into three broader domains. The most highly rated one was “Accept and forget,” followed by” Sadness and depression,” and “Physical and psychological aggression.”

Consistent with our initial prediction and the literature on rejection (Hartgerink et al., 2015), the “Sadness and depression” domain emerged, signifying that the staying partners would experience negative emotions, including sadness, depression, and disappointment. Emotions could motivate corrective actions, such as trying to persuade their partners to reconsider staying in the relationship and inquiring about the reasons for the relationship's dissolution, which is a probable reason why they loaded to this domain. There is also the possibility that being depressed may communicate to the departing partners the staying partners’ strong interest in them and the relationship. Since love and commitment are highly desirable in a mate (Buss, 2016), such communication may trigger second thoughts and prompt the departing partners to reevaluate their decision. Future studies should explore this possibility.

“Feeling sad” was the most probable response, followed by “Ask why,” with more than 92% and 84% of the participants, respectively, indicating that they would likely react in these ways. The “Spy on him/her” reaction also loaded in this domain, suggesting that, driven by their emotions, people would monitor their departing partners’ movements, possibly as a way to understand why they wish to terminate the relationship. The emotional distress would also lead individuals to seek the help of a psychologist. They might also suggest to their partners to visit a psychologist together, presumably as an attempt to salvage the relationship.

Also in line with our original prediction, the “Physical and psychological aggression” domain emerged, where staying partners would become angry and aggressive toward departing partners. Staying partners may also seek revenge sex by engaging in sexual activities with someone their partners know, as a means to inflict psychological pain on the departing partners. Threatening self-harm would also be another strategy to cause psychological distress to the departing partners and manipulate them into staying in the relationship. While more than 67% of the participants indicated they would likely become angry, the majority of participants deemed aggression, revenge sex, and threatening suicide as unlikely reactions.

Although not initially predicted, the “Accept and forget” domain emerged, where people would attempt to accept their partners’ decision to end the relationship and endeavor to leave the failed relationship behind. This would involve cutting all contact with their partners and engaging in activities or work to avoid dwelling on the relationship. “Occupying my mind with something else” and “Acceptance” were among the most likely reactions, with more than 81% and 75% of participants, respectively, indicating a high likelihood of responding in these ways. These reactions probably reflect coping strategies for dealing with the termination of the relationship.

Our prediction that the termination of the relationship would harm the self-esteem of the staying partners was not confirmed, as no such factor or domain emerged. Nevertheless, in Study 1, we did find the “My ego would be hurt” reaction, which, however, did not load onto any of the identified factors in the subsequent analysis. Moreover, we found reactions such as “I would feel down” loading to the “Feel sad” factor, which are likely to tap into self-esteem. The fact remains, however, that harm to self-esteem did not emerge as a separate factor, and we think the most plausible reason is the following: In the current study, we aimed to examine how people would react to the termination of a desirable relationship. Breaking up is an act of rejection and would hurt self-esteem (Abrams et al., 2005); however, this is a consequence of, and not a reaction to, the act of breaking up. That is, people would not think that they could react to the termination of a desirable relationship by lowering their self-esteem, which is why no such reaction emerged.

Moreover, we found that participants indicated that they would be more likely to experience seven or eight reactions, with the majority of the sample falling between four and nine. This finding suggests that people are unlikely to experience just one reaction or another, but are more likely to undergo several reactions. We propose that these responses may occur sequentially: Initially, people may experience negative emotions such as sadness and anger, which in turn trigger other responses, such as inquiring about the reasons for the relationship's failure. Based on this information, they may attempt to change their partner's mind, and if unsuccessful, they may cease all contact with their partner to alleviate emotional pain. Future research should aim to examine the timing of these identified reactions.

As mentioned in the introductory section, Davis et al. (2003) proposed 10 distinct reactions to breaking up, while our research identified thirteen such reactions. Three of the reactions suggested by Davis et al., including “anger/hostility/revenge,” “preoccupation,” and “interference with exploration,” closely align with three reactions we identified: “become aggressive,” “occupy my mind with something else,” and “ask why.” The remaining reactions proposed by Davis et al. did not emerge as distinct categories but instead loaded onto other categories we identified. For example, the reaction “self-blame” loaded under the “feel depressed” category in our study. The discrepancy between the factors proposed by Davis et al. and the one we identified underscores the importance of employing appropriate data reduction techniques. Conceptually identifying categories represents what researchers believe the reactions are, but factor analysis identifies reactions based on patterns in the data, making it more likely to reveal the true structure of reactions to the dissolution of an intimate relationship. Nonetheless, it is crucial to note that factor analysis is sensitive to the characteristics of the underlying sample, and additional replication studies are necessary to strengthen our confidence in the true factor structure.

Our prediction that men would tend to act more aggressively than women was not supported. For the “Become aggressive” reaction, no significant sex difference was found, and, in fact, the means were to the opposite direction of the predicted outcome: Women gave higher scores than men. One possibility is that the termination of a relationship may trigger “severe” aggression in men, which physically harms a partner, while in women, it may trigger “mild” aggression that intimidates but does not physically harm a partner. The “Become aggressive” factor includes items such as slapping and spitting on a partner, which are more closely associated with mild aggression, possibly explaining the observed mean difference. Future studies should aim to better distinguish between various aggressive responses and identify sex differences in them.

Older participants indicated that they were less likely to attempt to change their partner's minds and more likely to try to accept the decision to terminate the relationship and move on. One possible explanation is that, if leaving partners are persuaded to stay in the relationship, they may not be fully committed to it, making the relationship's prospects poor. Older individuals with more relationship experience may be more aware of this than younger ones, prompting them to accept the end of the relationship and move forward. Another possibility is that younger individuals experience romantic emotions such as love more strongly, which hinder them from moving on and motivate them to attempt to change their partner's mind. Future studies should investigate these possibilities further. Additionally, we did not detect any significant main effect of relationship status on any of the identified reactions.

We found that the termination of a desirable relationship would trigger negative responses such as aggression and self-harm. In some cases, these responses may escalate, with staying partners causing serious harm to their mates or themselves. More common responses include emotional pain, which would lead to more serious mental difficulties, such as depression. This emotional pain may also have positive future outcomes, as it would make people more cautious in selecting the right mate. However, in some instances, it may lead to future negative outcomes. For example, the emotional pain may motivate some individuals to exit the mating market in order to avoid experiencing such emotions again. As singlehood is typically associated with lower fitness than having an intimate partner, it can trigger negative emotions such as loneliness and sadness (Apostolou et al., 2019). In light of this, some people may choose to avoid relationships to escape the confrontation with these emotions. These arguments illustrate the complexity of human mating and underscore the importance of understanding how people react to the termination of an intimate relationship.

### Limitations and Future Directions

The current study aimed to compile an inclusive list of possible responses in scenarios where one partner wants to leave and the other wishes to stay in the relationship. Still, this is only the first step, and further research is needed to comprehend this intricate phenomenon fully. To begin with, more replication studies employing confirmatory factor analysis are necessary to strengthen our confidence in the true factor and domain structure of these reactions. Furthermore, as we used nonprobability samples, replication studies are essential to assess the applicability of our results to the broader population. Cultural factors may also influence different responses. For example, in some cultural settings, acting aggressively toward a partner may be more socially acceptable than in others. Consequently, future cross-cultural studies could aim to identify potential cultural variations in responses. Additionally, future studies could explore how leaving partners respond to the reactions of staying partners, such as whether they reconsider terminating the relationship.

Furthermore, the actual or suspected cause of the termination of the relationship is expected to impact the reactions of the remaining party. For instance, if people's partners leave them for someone else, this may trigger more anger than if they leave due to incompatibility. Such a scenario could also trigger jealousy (see Buss, 2002), an emotional reaction we have not identified here. A possible reason is that we did not specify the reasons for the relationship's termination. Future studies should extend our work by examining people's reactions in scenarios that vary according to the reasons for breaking up.

Our study was limited to examining the effects of sex, age, and relationship status on reactions to the termination of an intimate relationship. Still, many other individual differences are likely to influence people's reactions to relationship termination. These factors may include the duration of the relationship, the strength of the emotional bond with the partner, personality traits, differences in mate value between partners, prospects for finding another mate, the presence of children, and more. Future studies could examine the effect of these on the identified reactions. Moreover, in the current study, we did not measure sexual orientation, which could be another factor predicting the identified reactions. Thus, future studies need to examine whether heterosexual and nonheterosexual people react differently to the termination of an intimate relationship. Additionally, in the current research, we asked participants how they would react in the scenario of interest rather than how they had actually reacted in such scenarios. The rationale behind this is that reactions depend on various factors, and most individuals have likely not encountered this scenario many times. Therefore, asking participants about their past reactions may not provide as much insight into potential reactions as examining how people might react in the future. Nevertheless, future studies can enhance our understanding of the phenomenon by investigating how people have actually responded in scenarios of interest.

## Conclusions

Individuals may find themselves in an intimate relationship that they perceive as desirable, but their partners do not, and wish to terminate it. Our research suggests that the staying partners are likely to respond in three primary ways: They may experience sadness and depression, accept the situation and attempt to move on, and/or engage in physical and psychological aggression toward their partners. These reactions may manifest in the form of at least 13 more specific reactions, which in turn may manifest in at least 79 specific emotional and behavioral reactions. The three-level structure along with the diversity of reactions at each level underscores the complexity of the phenomenon. Consequently, this complexity highlights the need for further research to fully understand it.

## Supplemental Material

sj-docx-1-evp-10.1177_14747049241312231 - Supplemental material for How People React to the Termination of an Intimate Relationship: An Exploratory Mixed-Methods Study Supplemental material, sj-docx-1-evp-10.1177_14747049241312231 for How People React to the Termination of an Intimate Relationship: An Exploratory Mixed-Methods Study by Menelaos Apostolou, Isaias Taliadoros and Timo Juhani Lajunen in Evolutionary Psychology

## Appendix

The following 10 reactions were identified in Study 1 but were removed from the analysis in Study 2.

1. I would feel surprised
2. My ego would be hurt
3. I would react coldly
4. I would tell him/her that he/she will regret it
5. I would improve myself to show him/her what he/she missed
6. I would try to access his/her emails/messages
7. I would cry
8. I would turn to my family for support
9. I would consume substances (e.g., alcohol) to avoid thinking about it
10. I would say things to him/her to make him/her feel bad
