# Supplementary material for: How People React to the Termination of an Intimate Relationship: An Exploratory Mixed-Methods Study
Source: Evol Psychol. 2025 Jan 12;23(1):14747049241312231. doi: 10.1177/14747049241312231 (PMC11726518; doi:10.1177/14747049241312231)
Supplement: sj-docx-1-evp-10.1177_14747049241312231 - Supplemental material for How People React to the Termination of an Intimate Relationship: An Exploratory Mixed-Methods Study [file sj-docx-1-evp-10.1177_14747049241312231.docx]

**Supplementary material – the instrument to measure reactions to the termination of a desirable intimate relationship**

Instructions: Participants are provided with the following scenario: "You are in a romantic relationship that you would like to continue, and your partner tells you that he/she wants to break up. Please indicate how likely you are to react in the following ways using this scale: 1- Not at all likely to react this way, 5 – Very likely to react this way." All the items are scored in the following Likert scale: 1- Not at all likely to react this way, 5 – Very likely to react this way.

1. I would tell him/her not to make rash decisions and to think again
2. I would tell him/her to take some time to think it over
3. I would tell him/her that it is a shame to destroy everything we have built
4. I would ask him/her if there is room to change his mind
5. I would ask him/her if there is anything we can do to avoid getting there
6. I would tell him/her that I love him/her and I don't want to break up
7. I would ask him/her to give me a second chance
8. I would try to change his/her mind
9. I would ask him/her to discuss it
10. I would tell him/her that I am willing to make changes to make our relationship work
11. I would ask him/her if it is because of some problem that could be solved
12. I would ask him/her to consider the consequences of our separation for other people (family, children, friends)
13. I would show him/her how saddened I am by his/her decision
14. If we had, I would ask him to think about our children
15. I would cut off all contact with him/her
16. I would never speak to him/her again
17. I would block him/her on social media
18. I would disappear from his/her life
19. I would throw away/destroy anything that reminded me of him/her
20. I would try to get him/her completely out of my mind
21. I would respect his/her decision
22. I would accept his/her decision
23. I would accept it with dignity
24. I would wish him/her the best and move on
25. I would try to keep calm
26. I would accept his/her decision without doing anything
27. I would try to convince myself that it is better this way
28. I would self-criticize why we reached this result
29. I would get depressed
30. I would shut myself up
31. I wouldn't be in the mood for anything
32. I would be psychologically devastated
33. I would feel insecure
34. I would go crazy
35. I would have a hard time accepting that
36. I would blame myself
37. I would be disappointed
38. I would be shocked
39. I would be upset
40. I would slap him/her
41. I would become violent towards him/her
42. I would swear at him/het
43. I would throw his/her things on the street
44. I would spit on him/her
45. I would go out often to occupy my mind with something else
46. I would do activities to occupy my mind with something else
47. I would through in my work so I wouldn’t have to think about it
48. I would turn to my friends for support
49. I would have sex with others and make sure he/she found out
50. I would seek to have sex/relationship with someone he/she knows
51. I would seek to get into another relationship quickly
52. I would look for ways to get revenge on him/her
53. I would ask him/her to explain why
54. I would demand to know the reason
55. I would ask him/her if there is another person
56. I would try to understand the reasons why this happened
57. I would seek the help of a psychologist to overcome it
58. I would suggest to him/her that we see a psychologist
59. I would be angry
60. I would get irritated
61. I would feel rage
62. I would feel betrayed
63. I would be sad
64. I would feel down
65. I would be hurt
66. I would threaten him/her to kill myself
67. I would hurt myself
68. I would ask our mutual friends about his/her movements
69. I would monitor his/her social media movements

Scoring: The instrument is divided in 13 broad and three broader scales. For the 13 subscales calculate the mean score by averaging the relevant items. Change his/her mind: items 1-14, Cut all contact: items 15- 20, Acceptance: items 21-28, Feel depressed: items 29-39, Become aggressive: items 40-44, Occupy my mind with something else: items 45-48, Revenge sex: items 49 – 52, Ask why: items 53-56, Seek the assistance of a psychologist: items 57-58, Become angry: items 59-62, Feel sad: items 63-65, Threaten suicide: items 66-67, Spy on him/her: items 68-69. For the three subscale average the variables created in the previous step as follows: Sadness and depression, variables: Feel sad, Feel depressed, Change his/her mind, Ask why, Spy on him/her, Seek the assistance of a psychologist; Physical and psychological aggression, variables: Become aggressive, Revenge sex, Become angry, Threaten suicide; Accept and forget, variables: Cut all contact, Acceptance, Occupy my mind with something else
